# Retraction notice to: *Méningiome chordoïde extra-axiale: à propos d’un cas* (Pan Afr Med J. 2021 Feb 4;38:123)

**DOI:** 10.11604/pamj.2022.41.283.34682

**Published:** 2022-04-07

**Authors:** Pan African Medical Journal

**Affiliations:** 1 The Pan African Medical Journal, Yaoundé, Cameroun

**Keywords:** Retraction, case report, PAMJ

## Abstract

We hereby inform our readership of the retraction of the article “Méningiome chordoïde extra-axiale: à propos d´un cas” by Mohammed Guini, published in the Pan African Medical Journal 2021;38: 123 (doi: 10.11604/pamj.2021.38.123.20437).

*Access retracted manuscript*

## Retraction

After thorough investigation following a plagiarism complaint, it was concluded that Mohammed Guini is not the rightful author of this article. He did not participate in the management of the patient nor the writing of the article. This retraction complies with our policy on publishing ethics and integrity and the Committee on Publication Ethics (COPE) guidelines. The retracted article will remain online to maintain the scholarly record, but it will be digitally watermarked on each page as “Retracted”. The Pan African Medical Journal regrets that this serious breach of publication ethics was not identified before publication and sincerely apologizes to the scientific community.

## References

[1] Guini M (2021). Méningiome chordoïde extra-axiale: à propos d´un cas. Pan Afr Med J.

